# Insights and efforts to control rabies in Zambia: Evaluation of determinants and barriers to dog vaccination in Nyimba district

**DOI:** 10.1371/journal.pntd.0005946

**Published:** 2017-10-09

**Authors:** Carolyn Patricia Mulipukwa, Boyd Mudenda, Allan Rabson Mbewe

**Affiliations:** University of Zambia School of Medicine, Department of Public Health, Lusaka, Zambia; Wistar Institute, UNITED STATES

## Abstract

**Background:**

The current rabies control strategy in Zambia is based on dog vaccination, dog population control and dog movement restrictions. In Nyimba district of Zambia, dog vaccination coverage is low but the incidence of dog bites is high which places the community at risk of rabies infection. The renewed global interest eliminating rabies in developing countries has spurred interest to identify determinants and barriers of dog vaccination in an effort to reduce the overall disease burden.

**Methodology:**

A mixed methods cross sectional design was used in the study. This consisted of three parts: Evaluation of medical records regarding dog bite injuries, implementation and analysis of a household survey and in-depth review of key informant interviews. Data was collected into a Microsoft Excel database and subsequently transferred to STATA for descriptive, inferential and thematic analysis.

**Results:**

Dog vaccination coverage overall was 8.7% (57/655), with 3.4% (22/655) in urban areas, 1.8% (12/655) in peri-urban and 3.5 (23/655) in the rural regions. Financially stable households were more likely to have their dogs vaccinated. Only 10.3% (31/300) of the respondents had vaccinated their dogs and these had a reliable source of income as 6% (18/300) were peasant farmers, 2% (6/300) were dependants whose guardians were financially stable and 2.3% (7/300) were in steady employment. Important barriers to dog vaccination included cost, limited awareness of vaccination program and access.

**Conclusion:**

Current rabies control strategies in Nyimba district, Zambia, appear quite limited. Improvements in the regional dog vaccination program may provide benefits. Enhancement of educational efforts targeting behavioural factors may also prove useful. Finally, the cost of dog vaccination can be reduced with scaled up production of a local vaccine.

## Author summary

Dog vaccination against rabies is the main method of rabies control in Zambia and it is mainly conducted by the Department of Veterinary Services under the Ministry of Fisheries and Livestock. This study explores the factors influencing dog vaccination against rabies and the local barriers to rabies control in Nyimba district of Zambia. The study was done in three parts; a review of dog bite cases (n = 215 cases), a household survey and questionnaire (n = 300 households) and in depth interviews with local rabies experts (n = 5). The study found that the community had adequate knowledge about rabies prevention in dogs and humans. The area of residence, the age and financial capacity of the dog owner determined whether or not the household dog was vaccinated or not. The survey of the 300 households revealed that only 8.7% of the dogs were vaccinated against rabies and the dog bite case record review showed that the majority of the dog bite cases were caused by unvaccinated dogs most of which were owned. The study discusses the challenges in controlling rabies and possible reasons for non compliance to dog vaccination by the dog owners in Nyimba district.

## Introduction

Rabies has been a public health concern and has plagued both humans and animals since around 2000 BC [1]. The disease is endemic on all continents except Antarctica [2] and it is believed to cause approximately 59,000 human deaths annually [3]. Rabies is more prevalent in developing countries where management and control measures are poor; consequently, continents such as Asia and Africa have the highest incidence of rabies, accounting for over 95% of the global rabies cases [4].

Rabies can be transmitted between all warm-blooded species including man and studies have shown that several domestic and wild animals such as dogs, cats, cattle, wolves, foxes, jackals, bats and others can get infected with the rabies virus and transmit the disease to humans via bites or scratches. Human rabies is mostly due to dog-transmitted rabies virus (RABV) [5] which is an RNA virus of the Rhabdoviridae family genus *Lyssavirus* [6].

Following invasion of the central nervous system, rabies infection progresses rapidly [7] and death due to respiratory failure or cardiac arrest ensues [8]. While a very small number of patients with rabies have survived, the disease is untreatable and fatal once signs of encephalitis appear [9]. Fortunately, rabies post exposure prophylaxis (PEP) prevents rabies in humans exposed to the rabies virus [10]. Thus PEP is the cornerstone for rabies prevention in humans, and it is against this background that it is recommended for all persons that have been or suspected to have been exposed to the rabies virus.

In developing countries, the domestic dog has been found to be responsible for the transmission of most of the human rabies cases [3] with over 90% of the rabies cases being transmitted via dog bites [11]. It is estimated that owned dogs account for the majority of the hundreds of millions of people that are bitten by dogs in the world each year [2].

Cases of rabies transmission between humans through transplant surgery have been reported but these are very rare [12].

The control of rabies since 1973 as recommended by the World Health Organisation (WHO) includes; mass dog vaccination campaigns and strict dog population control via restricted breeding, restricted movements and culling of unwanted dogs especially stray dogs [13]. Although these control measures have been in place for about 50 years, studies have shown that only a few developed countries are currently rabies free [14]. The vaccination of dogs against rabies is now regarded as the most effective rabies control strategy combined with secondary roles of population control, movement regulations and promotion of responsible dog ownership [11] and [15].

In developing countries were the prevalence of rabies is still significantly high; dog vaccination is a challenge. A number of successful dog vaccination campaigns have been carried out and research has demonstrated that rabies in these countries can be controlled [16]. Studies have further shown that the common assumption that dog vaccination in developing countries is hindered by operational constraints such as lack of dog population knowledge, low public rabies knowledge and inadequate implementation resources, may be erroneous [17]. Dog vaccination may not be a priority in some developing countries because of the limited resources available [18].

In Zambia, rabies is regarded as one of the endemic scheduled or notifiable diseases and the law under the Zambian Animal Health (Control and Prevention of Animal diseases) Order of 2014 stipulates that "animal owners vaccinate their animals against all scheduled or notifiable diseases" [19]. The WHO recommends that vaccinating at least 70% of the dog population against rabies over consecutive years may interrupt rabies transmission chains amongst dogs [20]. It has been found that vaccination coverage lower than 30% of the dog population is a waste of resources [11].

In Zambia, the actual dog population is not well known but it is widely assumed that only a small percentage of the Zambian dog population is vaccinated against rabies. In Nyimba district for instance, the dog population estimate is based on the 2006 Livestock Census. According to the Zambian National Livestock Epidemiologic Information Centre (NALEIC) and District Veterinary records, only 5.4% (138/2,556) and 5.6% (157/2,804) of the estimated dog population were vaccinated against rabies in Nyimba district in 2013 and 2014 respectively.

Despite the estimated low number of vaccinated dogs in the country, the number of notified dog bite cases has continued to rise. According to the Zambian report on rabies presented at the Southern and East African Rabies Group (SEARG) meeting of 2013; the number of notified dog bite cases in Zambia rose from 620 in 2010 to 732 in 2011. Veterinary records in Nyimba district also show that there has been a steady increase in the number of notified dog bite cases from 84 recorded in 2013 to 134 cases recorded in 2014. The rise in dog bite cases has led to an increase in the demand for PEP in the district as most of the victims bitten by unvaccinated dogs require prophylaxis. The WHO estimates that the global annual cost of PEP to be around $1.6 billion [2]. This makes the use of PEP more expensive than simply vaccinating dogs against rabies.

Although a number of mass dog vaccination campaigns have been carried out in the district, the coverage has been very poor and the majority of the dog population remains unvaccinated. Thus both the dog and human population are at risk of rabies infection. An analysis of suspected rabies cases recorded in Zambia between 1985 to 2004 found 1,088 rabies positive samples from various species, 747 of which were from dogs and 98 were from humans (Table 1) [21]. Another analysis of brain samples collected from suspected rabid dogs between January 2005 and December 2013 found 153 rabies positive cases [22]. Thus the dog mediated human rabies and dog rabies burden in the district and the country at large is still a challenge.

**Table 1:** Rabies confirmed cases between 2004 and 2013 in Zambia.

Species	Dogs	Cattle	Human	Jackal	Total
Number of cases confirmed	747	139	98	24	1,088

Source: Munanang'andu et al., 2010 [21]

The aim of the study was to identify the socio demographic determinants which influence dog vaccination and the local barriers to dog vaccination against rabies. The study tried to explore the perceptions and responses of the dog-owning households in relation to rabies control in Nyimba district. It was hoped that gaining an understanding of the various social norms prevailing in the communities in relation to rabies control would help in the tailoring of vaccination campaigns which would result in a wider coverage.

## Methods

### Study area

The study was conducted in Nyimba district which is one of the nine districts of Eastern Province in Zambia (please follow this link: https://en.wikipedia.org/wiki/Eastern_Province,_Zambia#/media/File:Zambia_Eastern_Province_Districts.svg) [23]. The district lies on the southern part of the province and covers a total area of 10,509Km^2^ which is divided into valley and plateau areas. Nyimba district is situated approximately 340 Km from Lusaka the capital city of Zambia and 230 Km from Chipata the provincial centre of Eastern province.

According to the 2010 national population and housing census, the district has a human population of 85,025 with 60% of the population living in the plateau and 40% in the valley. A number of ethnic groups found in the district including the Nsenga, Chewa, Ngoni and Tumbuka with the Nsenga being the indigenous group. Agriculture is the main source of livelihood in terms of crop and livestock farming for about 90% of the population while the rest depend on fishing, casual labour and the civil service.

### Study design

**Fig 1:**
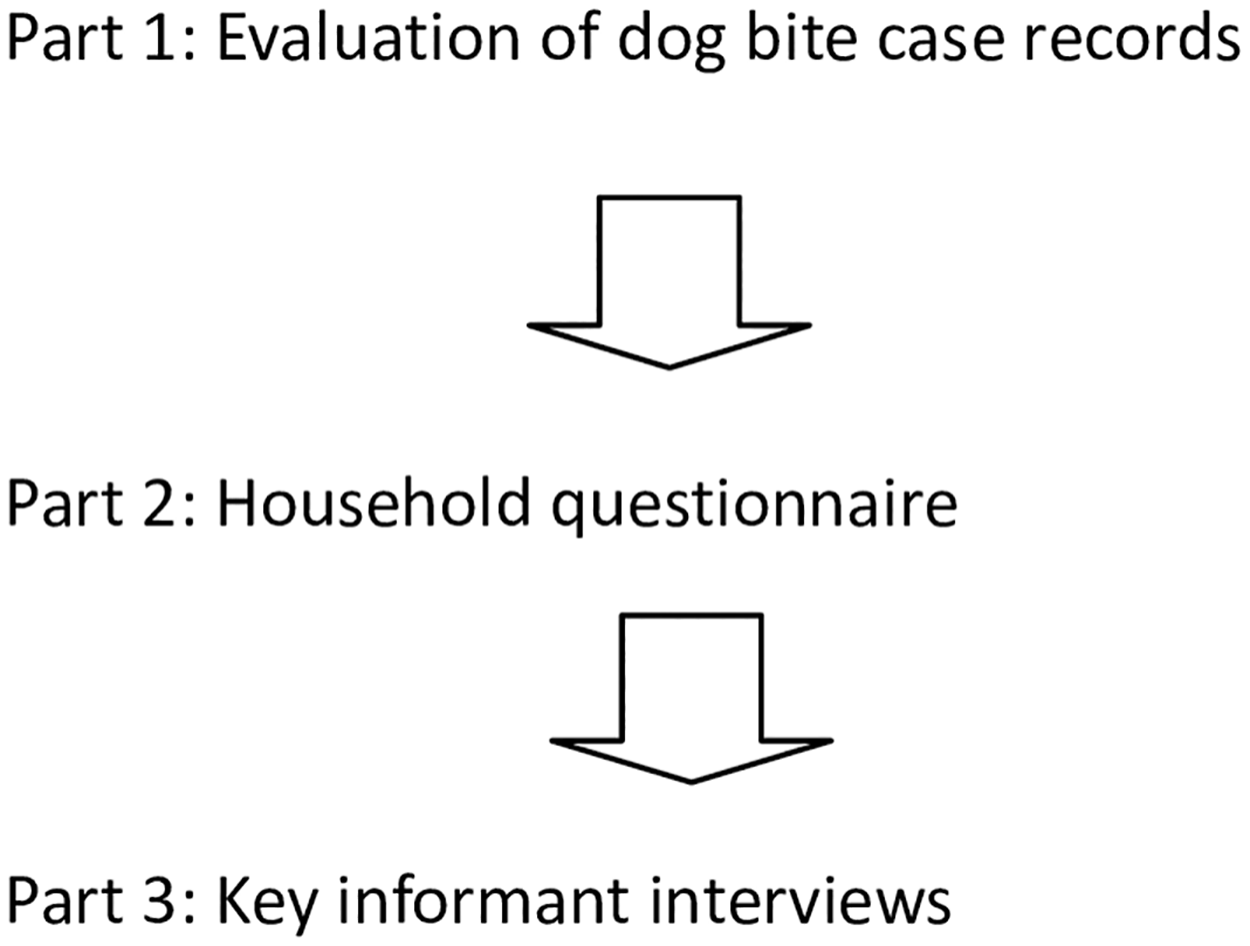
Parts of the study.

The research was conducted using a mixed methods (qualitative and quantitative) cross sectional design which was divided into three parts (Fig 1).

The first part consisted of evaluating dog bite case records at the veterinary office and Nyimba district Hospital to determine the frequency of dog bites and the vaccination and ownership statuses of the dogs involved in the bites. The proportion of reported dog bite cases which received rabies post exposure prophylaxis (PEP) from 2010 to 2013 was 12.3% (239/1,947). The postulated proportion of reported dog bite cases which received rabies PEP between 2013 and 2015 was hypothesized to be 5%. Calculations using statistical software at 0.05 significance level and 80% power showed that the required sample size was 130 reported dog bite cases. There were 215 dog bite cases recorded from 2013 to 2015 and they were all included in the study.

The second part was a survey of 300 households which responded to a household questionnaire which collected data on socio demographics, community knowledge with regards to rabies and data on dog population and vaccination coverage. The household survey sample was calculated using statistical software at 0.05 significance and 80% power. According to literature from the Central Veterinary Research Institute (CVRI) in Zambia, the relative prevalence of rabies in the country was hypothesized to be 39.7% and postulated to be 48%. The resulting sample size was 277 households which were rounded off to 300 households. The households were selected using a cluster randomized sampling method and the questionnaire was administered by the research assistants.

The third part included in depth interviews with local rabies experts including the rabies control officers from the Council and Veterinary Department of Nyimba district and the Central Veterinary Research Institute in Lusaka. The in depth interviews were conducted by the researcher.

### Ethics statement

For the in depth interviews with local rabies experts and the dog bite case record evaluation, verbal informed consent was obtained from the informers at both the hospital and the veterinary office. Written informed consent was obtained from respondents of the household questionnaire. Ethical clearance was obtained from Excellence in Research Ethics and Science (ERES) converge (Ref. No. 2015-June-018).

All data collection was conducted in English except for the household questionnaire which was done in Nsenga in some cases and then translated into English. Questionnaire and dog bite case evaluation data were entered into Microsoft Office Excel 2007 and coded then transferred to STATA version 12 for analysis. Data from the in depth interviews with local rabies experts was entered into Microsoft Office Word 2007 and thematic analysis was done manually.

## Results

### Evaluation of 2013 to 2015 dog bite case records

The evaluation of the dog bite case records found that there were 215 reported dog bite cases recorded between January, 2013 and January, 2015. Thus out of these 46% (99/215) were female and 53.95% (116/215) were male. Information on age was missing for 45% (98/215) of the cases but in 117 cases, the victims were aged between 1 and 68 years with a mean age of 16.8 years (SD 14.7). Approximately 62.4% (73/117) of the dog bite victims were aged between 1–15 years, 23.1% (27/117) were aged between 16–30 years and 14.5% (17/117) were over 30 years of age.

#### Characteristics of dog bite cases

Only 203 of the 215 reported dog bite cases were analysed (Table 2). The results showed that 72% (146/203) of the dog bite cases occurred in the rural areas of the district while 20% (41/117) occurred in the peri-urban and 8% (16/206) occurred in the urban areas. The study did not determine the circumstances that led to the bites but it was found that 46.8% (95/203) of the bites were caused by dogs whose owners were not known (considered to be stray) while 53.2% (108/203) of the cases were caused by dogs whose owners were known by the victim (considered to be owned). The immunization status of the stray dogs could not be determined, thus these dogs were considered unvaccinated. It was found that only 6.9% (14/203) of the cases were caused by vaccinated dogs while 93.1% (189/203) were caused by unvaccinated dogs. The vaccination statuses of the dogs involved in the bite cases were verified using veterinary records and dog rabies vaccination certificates. Only 6.9% (14/203) of the recorded cases produced valid dog rabies vaccination certificates which were consistent with the rabies vaccination records at the veterinary office.

**Table 2:** 2013 to 2015 dog bite case records evaluation.

Victims' area of residence	No. of dog bite cases	Offending dog status in terms of				Recommended therapy	
		Ownership		Vaccination			
		Known	Unknown	Vaccinated	Unvaccinated	PEP	Wound therapy
Urban	16	16	0	6	10	14	2
Peri-urban	41	23	18	6	35	34	7
Rural	146	69	77	2	144	143	3
Total	203	108	95	14	189	191	12

The rabies vaccination certificate was considered valid if the dog bite occurred before the due date for the next/booster vaccination. The study did not conduct any antibody testing on any of the dog sera to determine if the dogs were protected against rabies.

The district veterinary office recommended rabies post exposure prophylaxis (PEP) for 94% (191/203) of the cases. Wound therapy consisting of cleaning and dressing of the bite wound and tetanus prophylaxis were recommended for 5.9% (12/203) of the cases. The 191 cases received complete PEP because they were bitten by unvaccinated dogs which could have been potentially rabid. The nature of the contact ranged between category II (minor scratches and abrasions) and category III (single or multiple transdermal bites or scratches). The 12 cases received wound therapy and tetanus prophylaxis because they were caused by vaccinated dogs and in some cases the nature of the contact was considered to be category I (licks on intact skin).

### Household survey

The household survey was conducted in order to determine a more accurate estimate of the dog population and vaccination coverage and also to determine community knowledge levels and responses to rabies and its control strategies. A total of 300 households consisting of 1,970 people completed the household questionnaire. The average number of people per household was 6.57 and occupants ranged from 1 to 15 people in the households. Males made up 74% (223/300) of the respondents and 26% (77/300) were female. Two of the respondents were aged 14 years and they answered the questionnaire in homes where adults were not present at the time of the interview, the other 298 respondents were above 14 years of age (range 14–83 years, mean age = 38.6 years).

The spatial distribution of the 1,970 people covered by the survey was such that 78 (3.9%) were from the urban areas, 106 (5.4%) were from the peri-urban areas and 1, 786 (90.6%) were from the rural areas of the district.

As a means of livelihood it was found that peasant farming was practiced by 262 (87.3%) of the respondents while 15 (5%) were formerly employed and 23 (7.7%) were dependents. The results showed that of the 300 respondents, 49 (16.3%) did not have any formal education, 169 (56.3%) had Primary education, 78 (26%) had Secondary education and only 4 (1.3%) had Tertiary education.

#### Distribution of dog keeping households

Dogs are a common feature in the district and the study found that out of the 300 households surveyed, 256 (85.3%) kept dogs and only 44 (14.7%) did not. The distribution of dog keeping households was such that 13 (5%) were from the urban areas, 15 (5.8%) were from the peri-urban areas and 228 (89.1%) were from the rural areas of the district. It was observed that the number of dogs kept per household varied (range 1–12, mean = 2.2 dogs per household) depending on the area of residence and the perceived use of the dogs in the household.

Four categories of dog keeping households were identified based on the number of dogs kept (Table 3). Category I households kept 1–3 dogs and made up 202 (78.9%) of the dog keeping households. Category II households kept 4–6 dogs and made up 42 (16.4%), category III kept 7–9 dogs and made up 8 (3.1%) and category IV kept 10–12 dogs and made up 4 (1.6%) of the dog keeping households. Households falling in categories III and IV were only found in the rural areas of the district.

**Table 3:** Categories of number of dogs kept per household by area of residence.

Respondent area of residence	Categories of households					Total	Total people (%)
	I (1–3)	II (4–6)	III (7–9)	IV(10–12)	V (13–15)		
Urban	1 (2.9)	6 (5.4)	6 (4.8)	0	0	13	78 (3.9)
Peri-urban	5 (14.7)	3 (2.7)	7 (5.6)	1 (3.6)	1 (33.3)	17	106 (5.4)
Rural	28 (82.3)	101 (91.8)	112 (89.6)	27 (96.4)	2 (66.7)	270	1,786 (90.6)
Total (%)	34 (11.3)	110 (36.7)	125 (41.7)	28 (9.3)	3 (1.0)	300	1,970 (100)

A total of 655 dogs were found in the households giving a dog to human ratio of 33 dogs per 100 people (Table 4). The spatial distribution of the 655 dogs was such that 22 (3.3%) were from the urban areas, 35 (5.3%) were from the peri-urban and 598 (91.3%) were from the rural areas.

**Table 4:** Ratio of humans to dogs.

Residence for respondents	Distribution of humans in relation to dogs		Ratio of humans: dogs
	People (%)	Dogs (%)	
Urban	78 (3.9)	22 (3.3)	3.5: 1
Peri-urban	106 (5.4)	35 (5.3)	3: 1
Rural	1,786 (90.6)	598 (91.3)	2.99: 1
Total	1,970	655	3: 1

#### Dog vaccination coverage

It was found that the vaccination coverage was very low with only 57 (8.7%) of the dogs being vaccinated against rabies and 598 (91.3%) being unvaccinated (Table 5). The distribution of the 598 unvaccinated dogs was such that 575 (96%) were from the rural areas and 23 (4%) from peri-urban areas. All the 22 dogs from the urban areas were vaccinated against rabies. The study found that the rural households were more likely to have dogs which were not vaccinated against rabies and this was statistically significant (OR- 11.4, CI-1.8–71.4, P = 0.01). It was also found that people aged between 31–50 years were less likely to have dogs which were not vaccinated against rabies (OR-0.11, 95% CI- 0.02–0.5, P = 0.003). Members of this age group made up 48.8% of the peasant farmers and 86.7% of the formerly employed. This age group was also found to be composed of people who have attained different levels of education implying that they had a better appreciation of dog vaccination as they made up 47% of the people with primary education, 38% with secondary education and 50% of the people with tertiary education. The sex and religious background of the household decision maker were not found to play any role in the vaccination of the household dogs. There was also no statistically significant relationship between dog vaccination and the number of people in the household. There was no statistically significant association found between level of education, occupation of the dog owner and number of dogs in the household with vaccination of the household dogs.

**Table 5:** Dog vaccination status by area of residence.

Area of residence	Vaccination status of dogs n = 655		Total
	Vaccinated No (%)	Unvaccinated No (%)	
Urban	22 (39)	0	22
Peri-urban	12 (21)	23 (4)	35
Rural	23 (40)	575 (96)	598
Total	57 (8.7)	598 (91.3)	655

#### Community rabies knowledge

The respondents generally had a fairly good idea as to what rabies was and consequences of infection. The local name for rabies in Nsenga is *'Kambwambwa'* which means a disease of madness in dogs. Approximately 94.7% (284/300) of the respondents related rabies in humans and animals to loss of sanity which eventually led to death. At least 17.7% (53/300) did not know any symptoms of rabies but 82.3% (247/300) were able to list some symptoms of rabies in dogs (biting of inanimate objects, people and other animals, drooling and straying from home).

The transmission of rabies was linked to dog bites by 97% (291/300) of the respondents. The general belief was that rabies causes madness in dogs and once bitten by a rabid dog the victim also becomes mad. It was evident that rabies was greatly feared in the communities as 93.7% (281/300) of the respondents felt that all dog bites could potentially result in rabies infection so once a person was bitten it was important to seek help as soon as possible.

However, it was found that both conventional and traditional medicines were used when treating dog bites. When asked where dog bites could be treated, 93% (279/300) of the respondents mentioned the hospital or clinic. However, 32.3% (97/300) of the respondents mentioned traditional medicine as an alternative treatment to dog bites. The study found that the traditional method of treating dog bite wounds consisted of applying some herbs and some burnt dog hair from the offending dog onto the wound.

It was generally agreed that rabies could be prevented by vaccination of dogs by 280 (93.3%) of the respondents. Some of the respondents (2.3%) however, claimed that traditional methods such as piercing the dog's ears with hot iron bars, feeding fermented maize meal and cutting the tail could prevent rabies in dogs.

These methods were not offered as an alternative to dog vaccination by the investigators but rather were practised by some dog owners in the rural areas. There were at least 13 (4.3%) respondents who said they did not know how to prevent rabies in dogs.

According to 93% of the respondents, all dog owners had the responsibility to ensure that their dogs were vaccinated against rabies. Indeed, local bylaws stipulated that if a dog bite was caused by an unvaccinated dog, the owner of that dog had to cover the cost of the treatment for the victim. In fact, these dog bites are routinely reported to the police and the owners (if known) are instructed to pay for treatment. All dog owners who found themselves in this predicament subsequently paid for the victim's treatment. The actual number of dog owners who paid for PEP was not determined by the study. The high cost of treatment forced most of the dog owners to have their dogs vaccinated against rabies due to fear of having to pay for other cases in the future.

#### Local barriers to dog vaccination

It was quite clear that the respondents knew that they had to have their dogs vaccinated against rabies but a number of factors were found to prevent this from happening. The vaccination of dogs was perceived to be expensive by 56.3% of the respondents. It was found that dogs were rarely bought but given by friends or family hence only a few dogs were valued thus it was felt that it was not necessary to waste money on them. Some of the respondents suggested that rabies vaccination should be free to allow those who could not afford it to vaccinate their dogs.

The vaccination of dogs was also found to be hindered by the vastness of the district and the lack of adequate veterinary field staff to carry out the vaccinations. Interviews with the veterinary office revealed that the district had only 5 field officers; the district veterinary officer, livestock officer and three veterinary camp officers.

It was virtually impossible for these officers to cover the entire district given the high dog population and the limited resources available. Indeed 22% of the respondents said they had never met people from the veterinary office.

The limited number of veterinary field staff was found to have also affected the dissemination of dog vaccination information during vaccination campaigns. At least 21.3% of the respondents said that they did not know when dog vaccinations were being conducted in their communities. Interviews with the district veterinary staff revealed that rabies vaccination coverage was hindered by a number of operational constraints especially the lack of resources. The department relies on government funding which does not come regularly.

It was found that dog handling was a problem at the time of vaccination. Approximately 92% of the dog owners had difficulty controlling their dogs. In fact, veterinary control efforts were often thwarted by dog behaviour. Dog owners (83%) could not restrain their dogs and dogs often escaped when vaccination efforts were attempted.

#### Challenges in implementation of rabies control strategies

There was a general lack of enforcement of dog movement restriction in the district. Dogs were free to roam the streets and were frequently spotted scavenging for food at the dump sites, market area and the district hospital. Dog movement restriction was a challenge because only a negligible number of households were enclosed in wire, grass or wall fences and only a few dog owners managed to tie up their dogs. It was observed that there was no means of identifying owned and vaccinated dogs in the district except through dog vaccination certificates. The local council responsible for dog registration was also financially handicapped.

The council attributed the lack of dog registration and collaring to the cost of producing unique district collars to high cost of procurement which would make them unaffordable to most members of the community.

## Discussion

The research findings confirmed that dog bites cases were a common feature in the district. Although the study was not able to determine the proportion of unreported dog bite cases, it was assumed that the actual numbers were much higher. Despite the majority of survey respondents indicating that they would report dog bite cases to relevant authorities, studies have shown that underreporting of dog bite cases is common in most rabies prone communities. A study in Tanzania found that for every one case reported; at least 10 went unreported [18].

The lack of proper means of dog identification in the district made it difficult to accurately determine if a dog was owned or stray. However, it was evident that most of the cases are caused by owned dogs. A study in Kwa Zulu Natal of South Africa found that 89.7% of the dog bites were actually caused by owned dogs, 71% of which belonged to the neighbours of the victim [10]. Our study showed that owned dogs caused about 7% more dog bites than stray dogs although the actual figure is expected to be much higher.

This showed that there was a lack of compliance to rabies control regulations by the dog owners. Most of the developed countries are currently rabies free because of compliance with rabies control regulations. In Nyimba, most dog owners were only moved to vaccinate their dogs when their dogs were involved in dog bite cases and did not bother to restrict dog movement and breeding.

Enforcement of rabies control regulations was also found to be lacking in the district. The collaring and registering of owned dogs is supposed to be done by the district council. However, at the time of the survey, there was neither a registered nor collared dog in the district. Interviews with the Environmental Officer from the district council revealed that the cost of dog registration and collaring was almost ten times higher than the cost of vaccination (about K100.00 or $10.7 US dollars). The high cost was attributed to the production of the registration certificates and collars which could not be produced locally in the district. The certificates and collars are supposed to be engraved with a special seal and a unique district code to prevent easy reproduction of imitations.

Other problems included dog reproduction, movement and behavioural control issues. Enforcement of dog control was compromised by the environment—with few households having fenced property, allowing dogs to reproduce and move about unrestrained. Although the majority of the study population agreed that households could only keep a limited number of dogs, it was difficult to control dog movements and breeding which inadvertently increases the owned dog population.

The vaccination coverage was found to be 8.7%, far short of the WHO recommended 70% coverage rate. We found that the chances of dog vaccination were higher in the urban areas than in the rural regions of the district based on the survey. Indeed, dog bite case records showed a similar trend. This finding can perhaps be attributed to the different dog rearing techniques employed in the different residential set ups which of course are influenced by the livelihood patterns of the residents. A study in Tanzania found that dog-owning households in urban areas exhibited a closer human-to-dog relationship than their counterparts in the rural areas. The nature of this relationship was found to be dependent on the household livelihood patterns which in most cases determined the number of dogs kept in the household [18].

Peasant farming was found to be a common occupation in the district. Dog ownership appeared to be linked to that, as a form of security against crime. The dogs were also used to ward off crop damaging pests such as monkeys and rodents and also assisted in herding of livestock. As a result, the households in the rural areas were found to keep more dogs but were unable to care for them adequately due to the lack of interest in their wellbeing.

The urban households were found to keep a maximum of 3 dogs and all the dogs from this area found at the time of the study were vaccinated against rabies. Whether this phenomenon holds true in follow up studies remains to be determined. Dog vaccination in urban areas may be easier to achieve than in the rural areas due to the relative ease of access to vaccination information and vaccinators. The rural areas of the district are not easily accessed by the dog vaccinators mainly due to poor road networks, inadequate resources and geographically large areas to enable dog vaccination. Although the rural areas may have the highest dog population, they are not routinely targeted during dog vaccination campaigns.

The study found that the age and occupation of the household decision maker determined whether the household dog was vaccinated against rabies or not. According to the survey findings, the household heads falling in the 31–50 years age group were the most likely to have their dogs vaccinated against rabies. In terms of occupation, the majority of people in this age group were found to be actively involved in income generating activities hence they could afford to pay for dog vaccination. A working hypothesis of the study had proposed that households with fewer people would immunize their dogs at a higher rate because of the availability of resources, yet this was not shown. There was no statistically significant relationship between dog vaccination and the number of people in the household.

The survey showed that 97% of the respondents had adequate rabies transmission knowledge and this was found to be consistent with findings reported in other studies. Studies conducted in South Africa and Zimbabwe showed that 86% and 74% of the respective study populations had adequate knowledge in rabies transmission [10]. Our study did not seek to determine the source of rabies knowledge but it was assumed that rabies knowledge was disseminated by the veterinary department as was the case in both South Africa and Zimbabwe [10]. Our study showed that the study population was aware that dog vaccination could significantly reduce rabies transmission despite the low number of vaccinated dogs in the district.

Dog owners in the district attributed non compliance to dog vaccination to a number of local barriers. The cost of having a dog vaccinated was cited as a barrier by 56.3% of the study population. As pointed out by earlier studies, most rabies cases occur in resource poor communities which are characterised by poor dog ownership practices and unwillingness or inability to pay the full cost of vaccination resulting in low numbers of dogs being vaccinated [24].

Our study found that the cost of vaccinating a dog in Nyimba was K15.00 ($1.6) which was low compared to districts such as Lusaka the capital city of Zambia where dog vaccination cost between K25.00 to K50.00 ($2.6–$5.2). Although the cost of having a dog vaccinated at K15.00 ($1.6) is almost the same as the cost of two bottles of beer (K8.00 or $0.84 each) or a litre of gasoline (K12.50 or $1.31), in rural areas people drink illicit beer which costs around K3.00 ($0.31) and use bicycles for transport or walk. However, the inability to have dogs vaccinated against rabies could be a reflection of the value dog owners place on their dogs. In most circumstances, spending money on the household dog is considered a waste of resources.

The other reason for non compliance is that the vaccine was not usually available in the district. The availability of the rabies vaccine was dependent on the Veterinary Office, since there were no private veterinary clinics at the time of the study. Due to financial constraints, the veterinary department rarely had enough resources to have adequate stocks of the vaccine in the district. The price of rabies vaccine manufactured in South Africa had been fluctuating between K35.00 ($3.7) for 10 doses in 2013 to K75.00 ($7.9) in 2015. However, the Central Veterinary Institute (CVRI) in Lusaka locally produced rabies vaccine at a cost of K75.00 ($7.9) for 25 doses in 2015 but production was low. Thus each dose of the rabies vaccine from South Africa was K7.50 ($0.8) while that of the locally produced vaccine was K3.00 ($0.3).

The CVRI is the only vaccine producing institute in the country hence was unable to keep up with the demand as it had to supply to all the districts in the country. Lack of adequate resources severely restricted the production of local vaccine and the institution failed to meet the demand. Thus, the costs of dog immunization using the local product could be much reduced as more dogs could be vaccinated.

Low vaccination participation rates in dog vaccination campaigns were observed due to poor advertising and planning of the events. According to study findings, at least 17.6% of the dog-owning households said that they had not received relevant information when dog vaccinations were in progress. During dog vaccination campaigns, the dissemination of information of dog vaccination was done by the veterinary camp officers. Nyimba district only had 3 veterinary camp officers and they were unable to disseminate information to the remote parts of the district due to the lack of adequate fuel and the vast distances they had to cover. In the past, the veterinary assistants were assisted by community livestock assistants (CLAs) in terms of information dissemination, organisation of dog vaccination campaigns on the ground and community mobilisation on dog vaccination days.

Unfortunately this is no longer the case. The CLAs were community members trained by the veterinary department to carry out simple veterinary services such as spraying and deworming livestock in areas which did not have field officers. The work done by the CLAs was voluntary, however efforts to maintain the program were not sustained, and unfortunately these personnel were no longer able to assist the community.

Although information dissemination was found to be important, it was equally important that the information was targeted at recipients who were capable of acting on it. In most cases rabies control sensitisation meetings had no specific target audience. Efforts to attract participants to rabies control meetings seemed misguided—as music and drama were often employed—which catered to younger age groups and those not directly responsible for dog ownership and dog vaccination.

Although estimates of the dog population in Nyimba district are not well known, we believe the dog population is far greater than what the Livestock census in 2006 reported. Our survey results were surprising—showing that the dog-to-human ratio was 33 dogs per 100 people. This ratio is similar to that found in Cambodia [25] but much higher than what had been found in previous studies for instance; Jackman and Rowan (2007) found that dog-to-human population densities varied from 14.9 dogs per hundred people in rural Zambia to 15.8 dogs per hundred people in rural Tanzania [26] and [16] while in the case of Katmandu in Nepal it was 21.3 dogs per hundred people [27]. In South Africa and rural villages in Mexico it has been reported that there were more than 30 dogs per hundred people [28] and [29]. These studies have shown that high dog-to-human ratios are linked to high dog bite cases which increase the risk of rabies transmission.

The WHO found that the incidence of dog bites is highest in children aged below 14. In our study, the review of the dog bite case records showed that dog bites were most common in children of the age group 6–15 (about 45% of the cases) for the data available. This finding is not surprising as members of this age group engage in a lot of outdoor activities where the possibility of encountering dogs is high. According to the Zambian 2010 census of population and housing, this age group accounts for roughly 50% of the Nyimba population. In the district, dogs frequent garbage dumping sites scavenging for food. These sites have no form of barrier to prevent dogs from gaining access to them and they are located in areas where human traffic especially unsupervised children is high. At these sites children are regularly seen deliberately provoking and taunting the dogs as a form of amusement and as a result they are bitten by the dogs. In both rural and urban areas, most of the dog bites occur in the compounds where the children and dogs interact.

As a means of preventing possible rabies infection, PEP was recommended for the management of nearly all the cases caused by unvaccinated dogs. However, PEP is not always available in the district and when available it is only stocked in the district hospital which is located in the urban area. The majority of dog bite victims who require prophylaxis have the added costs of transportation which are significant. These additional costs can be reduced by vaccinating dogs. Studies have shown that PEP alone is more expensive than dog vaccination in terms of rabies control and may not be cost effective in the long run [30, 31, 32]. Our study did not determine the number of cases that received the recommended PEP but it is possible that some cases did not receive prophylaxis.

We found that in response to the significant number of dog bites, the communities in Nyimba district have formulated local by-laws were dog bite victims are financially compensated for medical costs by the owner of the dog if the dog was not vaccinated against rabies. However, the results of the study suggest that reporting of dog bites is motivated by both the desire for financial compensation and fear of rabies transmission. In most of the cases, a dog that bites people is suspected to be rabid and is killed immediately by the community. This is especially true were the dog involved is unknown (stray dog). The fact that some dogs are intentionally provoked, defending their puppies or their households is not considered. Evidence of this is seen in our study where 93.7% of the study population said that all dog bite cases should be treated as suspected rabid cases.

The killing of suspected rabid dogs by the community has made rabies surveillance difficult as possible rabies positive samples are destroyed before they can be collected for laboratory analysis and diagnosis. The presence of accurate and prompt diagnosis is essential for any disease surveillance program. Rabies diagnosis can be reliably made from brain samples taken after death and also from saliva, urine, and cerebrospinal fluid samples, but these are not as sensitive and reliable as brain samples [33]. In situations where a delay in proper assessment of the rabies risk may be encountered, euthanizing the suspected animal is often done to protect the community.

Rabies surveillance is also limited by the lack of diagnostic facilities. At the time of the study, there were only 3 laboratories equipped to diagnose rabies: Samora Machel School of Veterinary Medicine and Central Veterinary Research Institute in Lusaka and the Regional laboratory under the veterinary department in Southern Province of Zambia. The lack of adequate diagnostic facilities is a common feature in Southern Africa.

The SEARG report of 2013 indicated that between 2010 and 2012 Botswana diagnosed 0.0309% (1/3,234) rabies cases, 0.067% (8/11,959) in Zimbabwe and 0.075% (1/1,327) in Zambia. Studies have shown that poor surveillance systems result in delayed control interventions and can reduce chances of disease elimination [13].

### Conclusions

The control of rabies in Nyimba district remains challenging. Our study showed that compliance with dog control and dog vaccination against rabies was low. Certainly, there were a number of significant social and economic barriers identified.

We believe that interventions to reduce the impact of rabies need to include the local populations in order to maximise benefit. Dog vaccination rates could be improved with sustained efforts at improving dog behaviours, reducing reproduction rates, and increasing access to low cost vaccine. Educational efforts still have room for improvement as well. This study helped to elucidate some of the areas important for targeting efforts to reduce the morbidity of dog bites and the danger of rabies in this region.

## Supporting information

S1 Strobe Checklist (DOC)
