# Supplementary material for: Insights and efforts to control rabies in Zambia: Evaluation of determinants and barriers to dog vaccination in Nyimba district
Source: PLoS Negl Trop Dis. 2017 Oct 9;11(10):e0005946. doi: 10.1371/journal.pntd.0005946 (PMC5648261; doi:10.1371/journal.pntd.0005946)
Supplement: S1 Strobe Checklist — (DOC) [file pntd.0005946.s001.doc]

STROBE Statement—checklist of items that should be included in reports of observational studies

|  | Item No | Recommendation |
| --- | --- | --- |
| **Title and abstract** | 1 | (*a*) Indicate the study’s design with a commonly used term in the title or the abstract  In the methodology section of the abstract it is stated that a cross sectional study design was used. |
|  |  | (*b*) Provide in the abstract an informative and balanced summary of what was done and what was found  **Background:** The rabies control strategy in Zambia is based on dog vaccination, dog population control and dog movement restrictions. The coverage of dog vaccination against rabies is low but the incidence of dog bite injuries is in humans is high which places the community at risk of rabies infection.  **Objective**: To identify determinants and barriers of dog vaccination against rabies in Nyimba district of Zambia.  **Design:** A mixed methods cross sectional design was used in the study. This consisted of three parts: Evaluation of medical records regarding dog bite injuries, implementation and analysis of a household survey and in-depth review of key informant interviews.  **Results:** Dog vaccination coverage overall was 8.7% (57/655), with 3.4% (22/655) in urban areas, 1.8% (12/655) in peri-urban and 3.5 (23/655) in the rural regions. Financially stable households were more likely to have their dogs vaccinated. Only 10.3% (31/300) of the respondents had vaccinated their dogs and these had a reliable source of income as 6% (18/300) were peasant farmers, 2% (6/300) were dependants whose guardians were financially stable and 2.3% (7/300) were in steady employment. Important barriers to dog vaccination included cost, limited awareness of vaccination program and access.  **Conclusion**: Current rabies control strategies in Nyimba district, Zambia, appear quite limited. Improvements in the regional dog vaccination program may provide benefits. Enhancement of educational efforts targeting behavioural factors may also prove useful. Finally, the cost of dog vaccination can be reduced with scaled up production of a local vaccine. |
| Introduction | | |
| Background/rationale | 2 | Explain the scientific background and rationale for the investigation being reported  It is estimated that about 95% of human rabies deaths occur in Asia and Africa. The control of rabies in developing countries is mainly focused on the vaccination of dogs against the disease but coverage is very low. A number of successful dog vaccination campaigns have been carried out and research has demonstrated that rabies in these countries can be controlled. Studies have further shown that the common assumption that dog vaccination in developing countries is hindered by operational constraints such as lack of dog population knowledge, low public rabies knowledge and inadequate implementation resources, may be erroneous (17).  In Zambia, despite rabies being a scheduled or notifiable disease, only a few dogs are actually vaccinated against rabies. Furthermore, the numbers of dog bite injuries recorded in the country continue to rise annually. According to the Zambian report on rabies presented at the Southern and East African Rabies Group (SEARG) meeting of 2013; the number of notified dog bite cases in Zambia rose from 620 in 2010 to 732 in 2011. Veterinary records in Nyimba district also show that there has been a steady increase in the number of notified dog bite cases from 84 recorded in 2013 to 134 cases recorded in 2014. The rise in dog bite cases has led to an increase in the demand for rabies post exposure prophylaxis (PEP) in the district as most of the victims bitten by unvaccinated dogs.  The aim of the study was to identify the socio demographic determinants which influence dog vaccination and the local barriers to dog vaccination against rabies. The study tried to explore the perceptions and responses of the dog-owning households in relation to rabies control in Nyimba district. It was hoped that gaining an understanding of the various social norms prevailing in the communities in relation to rabies control would help in the tailoring of vaccination campaigns which would result in a wider coverage. |
| Objectives | 3 | State specific objectives, including any prespecified hypotheses  The objectives of the study were 1) Identify the socio demographic determinants which influence dog vaccination, 2) identify the local barriers to dog vaccination against rabies and 3) to estimate the dog vaccination coverage in the district. |
| Methods | | |
| Study design | 4 | Present key elements of study design early in the paper  The study was conducted using mixed methods (qualitative and quantitative) cross sectional design which was divided into three parts.  The first part consisted of evaluating dog bite case records at the veterinary office and Nyimba district Hospital to determine the frequency of dog bites and the vaccination and ownership statuses of the dogs involved in the bites.  The second part was a survey of 300 households which responded to a household questionnaire which collected data on socio demographics, community knowledge with regards to rabies and data on dog population and vaccination coverage.  The third part included in depth interviews with local rabies experts. |
| Setting | 5 | Describe the setting, locations, and relevant dates, including periods of recruitment, exposure, follow-up, and data collection  The study collected secondary data from Nyimba district veterinary and hospital records on dog bite injuries which occurred between 2013 and 2015. Primary data was collected through a household questionnaire which was administered to 3 different residential areas (Urban, Peri-urban and Rural) identified in Nyimba district. The household survey was conducted from June to September, 2016. |
| Participants | 6 | (*a*) *Cohort study*—Give the eligibility criteria, and the sources and methods of selection of participants. Describe methods of follow-up  *Case-control study*—Give the eligibility criteria, and the sources and methods of case ascertainment and control selection. Give the rationale for the choice of cases and controls  *Cross-sectional study*—Give the eligibility criteria, and the sources and methods of selection of participants  The study retrospectively identified dog bite injury patients. All cases that occurred between January, 2013 and January, 2015 were included in the study. The household questionnaire was administered to households that had resided in the district for at least 3 years prior to the study. Households were selected using a cluster randomised sampling method. The in depth key informant interviews were conducted with officers involved in rabies control from the Council and Veterinary Department of Nyimba district and the Central Veterinary Research Institute in Lusaka. |
|  |  | (*b*) *Cohort study*—For matched studies, give matching criteria and number of exposed and unexposed  *Case-control study*—For matched studies, give matching criteria and the number of controls per case |
| Variables | 7 | Clearly define all outcomes, exposures, predictors, potential confounders, and effect modifiers. Give diagnostic criteria, if applicable  The outcomes of interest in the study were: Recommendation of medical management following dog bite injuries and the vaccination of household dogs. The recommended management of a dog bite injury was either PEP or wound treatment and anti tetanus therapy. Household dogs were considered to be vaccinated against rabies if owners were able to produce valid rabies vaccination certificates which were consistent with the rabies vaccination records at the veterinary office. The determinants included; Sex, age and area of residence of the dog bite injury victim, the ownership and vaccination status of the dog involved in the dog bite injury case, the occupation, religious affiliation, level of education and household position of the respondent and the number of people and the number of dogs in the household. Potential confounders included age and sex of the dog bite injury victims. |
| Data sources/ measurement | 8* | For each variable of interest, give sources of data and details of methods of assessment (measurement). Describe comparability of assessment methods if there is more than one group  A dog was considered to be vaccinated if there was proof of a valid rabies vaccination certificate from the veterinary office. The course of management of reported dog bite injuries was shown by records of recommendations from the veterinary office and records of the management plan from the hospital. |
| Bias | 9 | Describe any efforts to address potential sources of bias  Selection bias could influence the association between the vaccination of the household dog against rabies and the area of residence of the dog owner. We compared the number of vaccinated dogs from the different areas of residence in the household survey to that involved in the dog bite injuries. Interviewer bias could influence the interviewers to select respondents who fit into the hypotheses of the study. We used a cluster randomized sampling method to select respondents. |
| Study size | 10 | Explain how the study size was arrived at  The sample size for the evaluation of the dog bite case records was calculated thus: The proportion of reported dog bite cases which received rabies post exposure prophylaxis (PEP) from 2010 to 2013 was 12.3% (239/1,947). The postulated proportion of reported dog bite cases which received rabies PEP between 2013 and 2015 was hypothesized to be 5%. Calculations using statistical software at 0.05 significance level and 80% power showed that the required sample size was 130 reported dog bite cases. There were 215 dog bite cases recorded from 2013 to 2015 and they were all included in the study.  The household survey sample was calculated using statistical software at 0.05 significance and 80% power. According to literature from the Central Veterinary Research Institute (CVRI) in Zambia, the relative prevalence of rabies in the country was hypothesized to be 39.7% and postulated to be 48%. The resulting sample size was 277 households which were rounded off to 300 households. |
| Quantitative variables | 11 | Explain how quantitative variables were handled in the analyses. If applicable, describe which groupings were chosen and why  In the evaluation of dog bite injuries age was grouped into 6 categories (1-5, 6-15, 16-20, 21-30, 31-50 and 51-70 years). The 117 case records that had complete information on age showed that the age of the victims ranged from 1-68 years and the mean age was 16.8 years (SD 14.7). The age of the respondents in the household survey was also grouped into 6 categories (15 years and below, 16-20, 21-30, 31-50 and 51 and above). The ages of the 300 respondents ranged from 14 to 83 years and the mean age was 38.6. The level of education of the respondents was grouped into 4 categories (No school, primary, secondary and tertiary). The results showed that at least 56% of the respondents had primary education. The number of dogs in the household was grouped into 4 categories (1-3, 4-6, 7-9 and 10-12). There were 655 dogs found in the 300 households. The number of dogs in the households ranged from 1-12 and the mean number of dogs per household was 2.2 dogs per household. The number of people per household was also grouped into 5 categories (1-3, 4-6, 7-9, 10-12 and 13-15 people). The total number of people found in the survey was 1,970. The number of people in the households ranged from 1-15 and the mean number of people per household was |
| Statistical methods | 12 | (*a*) Describe all statistical methods, including those used to control for confounding  The analysis of the dog bite injuries was limited to descriptive statistics which included: Counts, means, standard deviations and percentages. In the household survey, apart from the descriptive statistics, inferential statistics were carried out and the odds ratio was calculated using binary logistic regression, in identifying the determinants of vaccination of household dogs. |
|  |  | (*b*) Describe any methods used to examine subgroups and interactions  In the household survey, cross tabulations calculating Pearson's Chi square and Fisher's exact test were performed in tests of association between dog vaccination and the independent variables. After the multivariate regression, the likelihood ratio test was conducted to determine which variables produced the best the best fit model. |
|  |  | (*c*) Explain how missing data were addressed  There were no missing values in the household survey.  In the evaluation of dog bite injuries, we used the missing at random assumption (MAR) which allowed us to use multivariate imputation in STATA. We analysed the dog bite injury data with missing values suitably imputed in multivariate regression. The most likely value was estimated from the known values. However, the evaluation of the dog bite injuries was not the main focus of the study hence the results were not reported |
|  |  | (*d*) *Cohort study*—If applicable, explain how loss to follow-up was addressed  *Case-control study*—If applicable, explain how matching of cases and controls was addressed  *Cross-sectional study*—If applicable, describe analytical methods taking account of sampling strategy  Not applicable |
|  |  | (*e*) Describe any sensitivity analyses  Not applicable |

Continued on next page

| Results | | |
| --- | --- | --- |
| Participants | 13* | (a) Report numbers of individuals at each stage of study—eg numbers potentially eligible, examined for eligibility, confirmed eligible, included in the study, completing follow-up, and analysed  In the evaluation of dog bite injuries, the required sample size was 130. The records showed that 215 dog bite injuries had been reported between 2013 and 2015. The 215 cases were examined for inclusion in the study. Apart from missing age, only 202 cases had complete information and were eligible to take part in the study.  In the household survey, the required sample size was 277 however, 300 households were surveyed. Two respondents answered the questionnaire in households were adults were not present at the time of the survey. All 300 respondents were included in the survey and analysed. |
|  |  | (b) Give reasons for non-participation at each stage  In the evaluation of dog bite injuries, information on age of the victim was missing in 98 of the case records, thus only 117 cases were analysed for age. Since the eligible number of cases was below the required sample size, it was decided that the analysis done on age would not be conclusive. The case records had varying degrees of missing information: The area of residence were the dog bite occurred was missing in 9 cases, the recommended management of the dog bite injury was missing in 10 cases, the vaccination status of the dog involved in the bite was missing in 13 cases and in 12 cases there was no information to determine whether the dog involved was owned or not. At least 202 case records had the necessary information (except age) which allowed for descriptive analysis. |
|  |  | (c) Consider use of a flow diagram  Flow diagram of cases recruited in evaluation of 2013 - 2015 dog bite injuries.  Required number of dog bite injuries reported between 2013 and 2015 n=130  Assessed for eligibility  n= 215  **Excluded** (Total n=13). Missing information on:  -Residential area n=9  -Dog ownership status n=12  -Dog vaccination status n=13  -Recommended dog injury management n=10    Total recruited n= 202  **Data available for analysis**  -Residential area n=206  -Dog ownership status n=203  -Dog vaccination status n=202  -Recommended dog injury management n=205  -Sex n=215  -Age n=117  * Age of bite victim missing in 98 case records  Flow diagram of respondents recruited in household survey  Required number of Respondents for household questionnaire n=300  Assessed for eligibility n=300  **Excluded** n=0    Total recruited n= 300  **Data available for analysis**  -Residential area n=300  -Number of people in household n=300  -Number of dogs in household n=300  -Number of vaccinated dogs in household n=300  -Sex of respondent n=300  -Age of respondent n=300  -Respondents' education n=300  -Respondents' occupation n=300  -Respondents' religion n=300 |
| Descriptive data | 14* | (a) Give characteristics of study participants (eg demographic, clinical, social) and information on exposures and potential confounders  Table 1 Characteristics of dog bite injury victims   |  | Recommended dog injury management n=215 | | | | --- | --- | --- | --- | | Sex (%) | PEP | Wound therapy | Unknown | | Male | 105(48.8%) | 7(3.2%) | 4(1.9%) | | Female | 86(40%) | 12(5.6%) | 1(0.5%) | | Mean age (SD) | 7.7 (13.2) | 17.5 (20.2) | 14.4 (4.3) | |
|  |  | (b) Indicate number of participants with missing data for each variable of interest  Table 2 Characteristics of survey respondents   \|  \| Vaccination status of household dogs n=300 \| \| \| --- \| --- \| --- \| \| Sex (%) \| Vaccinated (%) \| Unvaccinated (%) \| \| Male \| 24 (8%) \| 199 (66.3%) \| \| Female \| 7 (2.3%) \| 70(23.3%) \| \| Mean age(SD) \| 40.4 (15.1) \| 38.2 (14.4) \| |
|  |  | (c) *Cohort study*—Summarise follow-up time (eg, average and total amount) |
| Outcome data | 15* | *Cohort study*—Report numbers of outcome events or summary measures over time |
|  |  | *Case-control study—*Report numbers in each exposure category, or summary measures of exposure |
|  |  | *Cross-sectional study—*Report numbers of outcome events or summary measures  Table 3 Summary measures of dog bite injuries   \| Area of residence \| No. dog bite injuries reported n=206 \| Dog ownership status n=203 \| \| Dog vaccination status  n=202 \| \| Recommended dog bite management n=205 \| \| \| --- \| --- \| --- \| --- \| --- \| --- \| --- \| --- \| \| owned \| stray \| Vaccinated \| Unvaccinated \| Wound therapy \| PEP \| \| Urban \| 16 (8%) \| 0 \| 16 (8%) \| 6 (3%) \| 9 (5%) \| 5 (2%) \| 11 (5%) \| \| Peri-urban \| 41 (20%) \| 18 (9%) \| 23 (11%) \| 6 (3%) \| 35 (17%) \| 7 (3%) \| 34 (17%) \| \| Rural \| 169 (82%) \| 77 (38%) \| 69 (34%) \| 2 (1%) \| 144 (71%) \| 6 (3%) \| 143 (70%) \|   Table 4 Summary measures of household survey   \| Area of residence \| No. of people n=1,970 (%) \| No. dogs in area n=655 (%) \| No. vaccinated n=57 (%) \| No. unvaccinated n=598 (%) \| \| --- \| --- \| --- \| --- \| --- \| \| Urban \| 78 (4%) \| 22 (3.3%) \| 22 (39%) \| 0 \| \| Peri-urban \| 106 (5%) \| 35 (5.3%) \| 12 (21%) \| 23 (4%) \| \| Rural \| 1,786 (91%) \| 598 (91.3%) \| 23 (40%) \| 575 (96%) \|   Table 5 Summary of dog vaccination in household survey   \| Variable \| Not vaccinated \| Vaccinated \| \| --- \| --- \| --- \| \| **Area of residence** \|  \|  \| \| Urban \| 0 \| 13 (41.9) \| \| Peri-urban \| 11 (4.1) \| 6 (19.3) \| \| Rural \| 258 (95.9) \| 12 (38.7) \| \| **Sex distribution** \|  \|  \| \| Male \| 199 (74) \| 24 (77.4) \| \| Female \| 70 (26) \| 7 (22.6) \| \| **Age of respondents in years** \|  \|  \| \| <15 \| 4 (1.5) \| 0 \| \| 16 - 20 \| 25 (9.3) \| 2 (6.4) \| \| 21 - 30 \| 59 (22.0) \| 7 (22.5) \| \| 31 - 50 \| 131 (48.7) \| 11 (35.5) \| \| 51 - 70 \| 42 (1563) \| 11 (35.5) \| \| 71 - 85 \| 8 (3) \| 0 \| \| **Level of education** \|  \|  \| \| No education \| 49 (18.2) \| 0 \| \| Primary \| 160 (59.5) \| 9 (29.0) \| \| Secondary \| 56 (21.6) \| 20 (64.5) \| \| Tertiary \| 2 (0.7) \| 2 (6.4) \| \| **Occupation status** \|  \|  \| \| Farmer \| 244(90.7) \| 16 (51.6) \| \| Dependant \| 17 (6.3) \| 6 (19.3) \| \| Employed \| 8 (3) \| 7 (22.6) \| \| **Number of people in household** \|  \|  \| \| 1-3 \| 29 (10.8) \| 5 (16.1) \| \| 4-6 \| 99 (36.8) \| 11 (35.5) \| \| 7-9 \| 112 (41.6) \| 13 (41.9) \| \| 10-12 \| 27 (10.0) \| 1 (3.2) \| \| 13-15 \| 2 (0.7) \| 1 (3.2) \| \| **Number of dogs in household** \|  \|  \| \| 1-3 \| 176 (65.4) \| 26 (83.9) \| \| 4-6 \| 38 (14.1) \| 4 (12.9) \| \| 7-9 \| 8 (3.0) \| 0 \| \| 10-12 \| 4 (1.5) \| 0 \| |
| Main results | 16 | (*a*) Give unadjusted estimates and, if applicable, confounder-adjusted estimates and their precision (eg, 95% confidence interval). Make clear which confounders were adjusted for and why they were included  Table 6 Main results for household survey   | Variable | Unadjusted | | | Adjusted | | | | --- | --- | --- | --- | --- | --- | --- | | Odds ratio | p-value | 95% CI | Odds ratio | p-value | 95% CI | | **Area of residence** |  |  |  |  |  |  | | Urban (Ref) |  |  |  |  |  |  | | Peri-urban | 11.0 | <0.0001 | 3.7 - 37.1 | 11.4 | 0.01 | 1.8 - 71.4 | | Rural | omitted |  |  | omitted |  |  | | **Age of respondents in years** |  |  |  |  |  |  | | <15 (ref) |  |  |  |  |  |  | | 16 - 20 | 0.3 | 0.1 | 0.1 - 1.5 | 0.11 | 0.3 | 0.003-5.1 | | 21 - 30 | 0.4 | 0.1 | 0.2 - 1.3 | 0.2 | 0.07 | 0.03-1.2 | | 31 - 50 | 0.3 | 0.014 | 0.1 - 0.8 | 0.11 | 0.003 | 0.02-0.5 | | 51 - 70 | omitted |  |  | Omitted |  |  | | 71 + | omitted |  |  | Omitted |  |  | | **Level of education** |  |  |  |  |  |  | | No education (Ref) |  |  |  |  |  |  | | Primary | 0.06 | 0.006 | 0.007 - 0.4 | 0.1 | 0.2 | 0.002 - 4.6 | | Secondary | 0.34 | 0.3 | 0.04 - 2.6 | 0.3 | 0.5 | 0.006 - 10.5 | | Tertiary | 0mitted |  |  | omitted |  |  | | **Occupation status** |  |  |  |  |  |  | | Farmer (Ref) |  |  |  |  |  |  | | Dependant | 5.4 | 0.002 | 1.9 - 15.2 | 7.3 | 0.4 | 0.1 - 531.8 | | Employed | 13.3 | <0.001 | 4.3 - 41.4 | 0.5 | 0.6 | 0.02 - 11.8 | | **Number of dogs per household** |  |  |  |  |  |  | | 1 - 3 | 6.3 | 0.07 | 0.8 - 48.1 | 7.6 | 0.1 | 0.6 - 90.2 | | 4 - 6 | 4.5 | 0.2 | 0.4 - 42.3 | 5.7 | 0.2 | 0.4 - 88.3 | | 7 - 9 | omitted |  |  | Omitted |  |  | | 10 - 12 | omitted |  |  | omitted |  |  | | **Number of people per household** |  |  |  |  |  |  | | 1 - 3 (Ref) |  |  |  |  |  |  | | 4 - 6 | 0.6 | 0.4 | 0.2 - 2 | 0.6 | 0.6 | 0.08 - 4.3 | | 7 - 9 | 0.7 | 0.5 | 0.2 - 2 | 0.6 | 0.6 | 0.09 - 4.5 | | 10 - 12 | 0.2 | 0.2 | 0.02 - 1.9 | 0.3 | 0.3 | 0.01 - 4.5 | | 13 - 15 | 2.9 | 0.4 | 0.2 - 38 | 0.8 | 0.9 | 0.01 - 92.0 | | **Sex distribution of respondents** |  |  |  |  |  |  | | Male (Ref) |  |  |  |  |  |  | | Female | 0.8 | 0.7 | 0.3 - 2 | 0.4 | 0.5 | 0.02 - 5.9 | |
|  |  | (*b*) Report category boundaries when continuous variables were categorized  Table 7 Continuous variable boundaries in household survey   \| Variable \| Range \| Mean \| Observations \| \| --- \| --- \| --- \| --- \| \| Age \| 14 - 83 \| 38.5 \| 300 \| \| Number of dogs per household \| 0 - 12 \| 2.2 \| 655 \| \| Number of people per household \| 1 - 15 \| 6.6 \| 1, 970 \| |
|  |  | (*c*) If relevant, consider translating estimates of relative risk into absolute risk for a meaningful time period  Not relevant |
| Other analyses | 17 | Report other analyses done—eg analyses of subgroups and interactions, and sensitivity analyses  Not applicable |
| Discussion | | |
| Key results | 18 | Summarise key results with reference to study objectives  The study found that the incidence of dog bite injuries was quite high in the district with 215 cases reported between 2013 and 2015. The coverage of dog vaccination against rabies was found to be significantly low (8.7%) which resulted in almost all (94%) dog bite injuries requiring rabies post exposure prophylaxis (PEP).  Enforcement of rabies control regulations was found to be almost non-existent with lack of compliance to dog registration, movement and breeding restrictions and vaccination against rabies. Although a number of barriers to dog vaccination were identified, dog owners aged between 31- 50 years and dwelling in the urban areas of the district were associated with dog vaccination.  We had hypothesised that households with fewer numbers of people and dogs would be associated with dog vaccination but this was not shown. Another hypothesis was that higher levels of education would influence dog vaccination but this was also not shown. |
| Limitations | 19 | Discuss limitations of the study, taking into account sources of potential bias or imprecision. Discuss both direction and magnitude of any potential bias  It has been observed that chances of children attending health facilities for medical attention are higher than that of adults. Our study may have underestimated the occurrence of dog bite injuries in adults as these cases are not commonly reported. Some studies have found that under reporting of dog bite injuries is quite high.  Peasant farming was found to be the main occupation among the respondents. It was assumed that this occupation did not generate adequate income. We may have underestimated the social economic status of the respondents the claim of the cost of dog vaccination being unaffordable maybe a reflection of how much dogs are undervalued by their owners.  The study did not determine the actual number of dog bite injuries that received the recommended PEP. Therefore, it is not possible to say whether adequate stocks of PEP were available given the high number of dog bite injuries recorded. |
| Interpretation | 20 | Give a cautious overall interpretation of results considering objectives, limitations, multiplicity of analyses, results from similar studies, and other relevant evidence  The overall dog vaccination coverage in the district is very low. This is not surprising given that dog vaccination is mainly concentrated in the urban area which accounts for about 3% of the dog population in the study. The findings are however not conclusive given the small sample size and possible selection bias. There is need for more research on the subject. |
| Generalisability | 21 | Discuss the generalisability (external validity) of the study results  The characteristics of the respondents in our study were generally typical for most developing countries. Most of the rural districts engage in peasant farming for livelihood and have limited access to health and education facilities. Some findings in our study such as the availability of community rabies knowledge, the human to dog population ratios and the operational constraints in the control of rabies are consistent with findings in previous studies from other developing countries. Rabies control has been and still remains a challenge in Africa. |
| Other information | | |
| Funding | 22 | Give the source of funding and the role of the funders for the present study and, if applicable, for the original study on which the present article is based  The study was funded by the Ministry of Fisheries and Livestock under the department of Veterinary Services. The ministry did not play any role in the selection of the study design, collection of data and analysis, drafting of the manuscript or decision to publish. The funding provided was used for logistics in data collection and compilation. |

*Give information separately for cases and controls in case-control studies and, if applicable, for exposed and unexposed groups in cohort and cross-sectional studies.

**Note:** An Explanation and Elaboration article discusses each checklist item and gives methodological background and published examples of transparent reporting. The STROBE checklist is best used in conjunction with this article (freely available on the Web sites of PLoS Medicine at http://www.plosmedicine.org/, Annals of Internal Medicine at http://www.annals.org/, and Epidemiology at http://www.epidem.com/). Information on the STROBE Initiative is available at www.strobe-statement.org.
